# The IL-17/IL-23 Axis and Its Genetic Contribution to Psoriatic Arthritis

**DOI:** 10.3389/fimmu.2020.596086

**Published:** 2021-01-07

**Authors:** Matteo Vecellio, Vivien Xanath Hake, Connor Davidson, Maria Cristina Carena, B. Paul Wordsworth, Carlo Selmi

**Affiliations:** ^1^ Nuffield Department of Orthopaedics, Rheumatology and Musculoskeletal Sciences, University of Oxford, Oxford, United Kingdom; ^2^ Division of Rheumatology and Clinical Immunology, Humanitas Clinical and Research Center, IRCCS, Milan, Italy; ^3^ Leukocyte Biology Unit, Humanitas Clinical and Research Center, IRCCS, Milan, Italy

**Keywords:** genetics, psoriatic arthritis, IL17, IL23, SNPs (single nucleotide polymorphisms), therapy

## Abstract

Psoriatic arthritis (PsA) is a chronic inflammatory disease belonging to the family of spondyloarthropathies (SpA). PsA commonly aggravates psoriasis of the skin and frequently manifests as an oligoarthritis with axial skeletal involvement and extraarticular manifestations including dactylitis, enthesitis, and uveitis. The weight of genetic predisposition to psoriasis and PsA is illustrated by the concordance rates in monozygotic twins which clearly demonstrate that genomics is insufficient to induce the clinical phenotype. The association of PsA with several single nucleotide polymorphisms (SNPs) at the IL23R locus and the involvement of Th17 cells in the immunopathogenesis of PsA clearly put the IL-23/IL-17 axis in the spotlight. The IL-23 and IL-17 cytokines have a pivotal role in the chronic inflammation of the synovium in PsA and are also prominent in the skin lesions of those with PsA. In this review, we focus on the genetic association of the IL-23/IL-17 axis with PsA and the contribution of these master cytokines in the pathophysiology of the disease, highlighting the main cell types incriminated in PsA and their specific role in the peripheral blood, lesional skin and joints of patients. We then provide an overview of the approved biologic drugs targeting the IL-23/IL-17 axis and discuss the advantages of genetic stratification to enhance personalized therapies in PsA.

## Introduction

Psoriatic arthritis (PsA) is a common inflammatory disease affecting the joints and it is usually accompanied by plaque psoriasis (Ps) (1). PsA occurs in up to 30% of patients with psoriasis (particularly those with nail involvement) and affects from 0,05 to 0,25% of the general population (2), making it the second most common form of chronic inflammatory arthritis after rheumatoid disease. To address the therapeutic choices and to envision the potential musculoskeletal and dermatological phenotypes, the GRAPPA (Group for Research and Assessment of Psoriasis and Psoriatic Arthritis) group identified six disease domains, i.e. peripheral arthritis, enthesitis, dactylitis, axial involvement, skin and nail psoriasis. Among these, peripheral arthritis and enthesitis are dominant and found in the vast majority of patients while the prevalence of axial PsA increases with disease duration, occurring in less than 5% of early referrals and up to 25–70% of patients with long-term disease course for PsA (2). PsA is in some cases characterized by axial skeletal involvement, along with the more frequent oligoarthritis with mostly peripheral and asymmetric manifestations (3).

The pathogenetic link between psoriasis and PsA is highly representative of the mechanistic hypotheses of disease pathogenesis. Psoriatic skin is featured by hyperplasia of the epidermis and of the stratum corneum, infiltration of the epidermis by neutrophilic granulocytes (called Munro’s micro abscesses) and infiltration of the dermis by T-cells, dendritic cells (DCs), and macrophages, which leads to the clinical features of raised erythematous silvery plaques (4). In a similar fashion, PsA is characterized by chronic inflammation which causes bone erosion and bone loss, but also new bone formation around the affected joints (5).

The increased number of osteoclasts found in the synovium in PsA is remarkably similar to rheumatoid arthritis (RA) and the persistent inflammatory synovitis causes progressive joint damage due to synovitis and erosion of articular cartilage, visible as radiological damage in almost half of the patients (6). The exaggerated inflammatory response lead to enthesitis, with the crucial contribution of IL-17 producing T-cells and enthesal resident cells, expressing IL-23R (7, 8) with biomechanical stress, HLA-B27, and a permissive microbiome as necessary factors (9).

The phenotypic features of PsA suggest that some of the genetics and molecular mechanisms of the disorder are shared across various different types of inflammatory diseases, including psoriasis, ankylosing spondylitis (AS), inflammatory bowel disease, and Behçet disease (10–12). Extra-articular manifestations of PsA include inflammation of the gastrointestinal tract (with a higher risk of inflammatory bowel disease) and the eye (uveitis), along numerous metabolic and neoplastic disturbances (13).

In this review we will address the contribution of genetics to susceptibility to PsA and its subsequent progression, with special emphasis on the IL-23/IL-17 axis and the genes and cells that this involves. We are well aware that genetics represents only a necessary but insufficient player in the disease etiology, as recapitulated by the low concordance rates in monozygotic twins for PsA. Of note, however, the same rates are for psoriasis among the highest in chronic inflammatory or autoimmune diseases.

## PsA Diagnosis

The diagnosis of PsA is largely based on features and the CASPAR criteria (14) are used in the research setting for more formal; classification purposes. In contrast to rheumatoid arthritis there are no specific markers or autoantibodies for the diagnosis of PsA: it is seronegative for both rheumatoid factor and antibodies to cyclic citrullinated peptides (CCP) unlike rheumatoid arthritis where these are positive in approximately 80% of cases. Overall PsA is equally distributed between males and females but axial manifestations (spondylitis) are about three times more common in males (15). PsA patients experience substantial functional impairment, decreased quality of life and reduced life expectancy, which highlights the importance of prompt implementation of appropriate treatment. Genetics and molecular studies have led to a better understanding of the etiology of the disease and in future may allow the development of more targeted individual approaches to therapy.

Laboratory tests typically but not invariably show increased raised inflammatory markers, such as erythrocyte sedimentation rate (ESR) and C-reactive protein (CRP), whereas rheumatoid factor (RF) and CCP antibodies are negative, in contrast to rheumatoid arthritis (16). Recent studies have also shown a possible association with anti-LL37 (cathelicidin antimicrobial peptide) autoantibodies (17).

There is an association with the Human Leukocyte Antigen (HLA)-B27 that is most prevalent in those with sacroiliitis and axial skeletal involvement. Of particular interest, about 85% of PsA patients with bilateral sacroiliitis are positive for HLA-B27 in contrast to only 22% of cases with unilateral sacroiliitis (18, 19). Conversely, HLA-B17/Cw6 haplotype (strongly associated with psoriasis itself) is mostly associated with oligoarthritis (20).

HLA-B27 is the key genetic marker of ankylosing spondylitis (AS), commonly shared with axial-PsA (21). Although the prevalence of HLA-B27 is lower in patients with PsA (20%), it has been demonstrated that axial-PsA patients are significantly HLA-B27 positive compared to patients without axial involvement (P < 0.001) (22, 23).

Radiological imaging of PsA patients may show signs of both bone erosion and new bone formation with bone proliferation mostly found in the metacarpal and metatarsal bones and joints. Formation of new bones is mostly asymmetric, with deformities arising at digits and peripheral joints. Joint damage affects mostly the hands, wrists, feet, ankles, knees, and shoulders. Syndesmophytes may develop in the skeleton and calcification may appear at the entheses, specifically at the insertion sites (24). In contrast to rheumatoid arthritis, PsA may show different manifestations in the same anatomical site with osteolysis and bone deposition detected in the same hand. Dactylitis may be accompanied by bone erosion or new bone formation as well (25). In the spine the degree of new bone formation can be graded using scoring systems, such as the Bath Ankylosing Spondylitis Radiology Index (BASRI) and the modified Stoke Ankylosing Spondylitis Spine Score (mSASSS) (26, 27).

## The Genetics, Epigenetics, and Immunopathogenesis of PsA

There is a strong genetic component to psoriasis and PsA. Further, there is a significant degree of overlap in genetic predisposition between psoriasis, PsA, AS and the inflammatory bowel diseases (ulcerative colitis and Crohn Disease) (21, 28).

The genetic contribution to diseases like psoriasis and PsA has classically been investigated through twin studies. The concordance rate for psoriasis in MZ twins is between 20 to 64%, indicating that genetic factors account for roughly 70% of the population variance in the susceptibility to psoriasis (18). In polygenic diseases, there has also been an increasing focus on monozygotic (MZ) twins in order to assess the influence of epigenetics. This is true also for psoriasis and PsA (29). In addition to genetic predisposition, the onset and progression of both psoriasis and PsA appear to be influenced by both the environment and epigenetics factors (30).

### Genome-Wide Association Studies and Array-Based Technologies

PsA is a polygenic immune-mediated disease: genome-wide association studies (GWAS) have identified many genes/genomic loci increasing susceptibility for PsA, many of which are also common to psoriasis uncomplicated by arthritis; these include *HLA-A*, *HLA-B*, *HLA-C*, *IL23R*, *CSF2* (Colony Stimulating Factor 2 or granulocyte-macrophage colony stimulating factor), *TRAF3IP2* (TRAF3 Interacting Protein 2), *NOS2* (Nitric Oxide Synthase 2) (31, 32). The results of GWAS suggest that there are PsA-specific genetic variants which are independent of those previously identified in isolated psoriasis, specifically near *IL23R* and *TNFAIP3* (TNFα Induced Protein 3) (33).

GWAS are very informative for common and low frequency variants but they do not identify rare variants. Exome chips, such as the Illumina Exome BeadChip, allow the identification of coding variants and detection of rare SNPs (34). A very good alternative is the ImmunoChip, such as the Illumina Infinium genotyping chip, which contains 196,524 polymorphisms (718 small insertion deletions, 195,806 SNPs) and it is a “low-cost” option. It is designed to perform deep replication of major inflammatory and autoimmune diseases and fine mapping of established GWAS significant loci (35). In 2015, Bowes and colleagues used the ImmunoChip array to fine-map immune-related susceptibility loci including the known psoriasis risk loci, to define new PsA susceptibility loci. They identified a specific PsA variant at the *IL23R* locus, and a new PsA-specific association at chromosome 5q31 (36).

These associations may indicate roles for certain signaling pathways that are specific to the pathogenesis of PsA rather than psoriasis itself, but further investigation is needed to clearly understand their contribution.

### PsA-Associated Genetic Variants and Their Immune-Pathological Role

Antigen presentation by MHC proteins is pivotal to acquired immunity, and MHC alleles are strongly associated genetically with both psoriasis and PsA. HLA class I alleles are associated with increased susceptibility for PsA, but the PsA-associated HLA alleles differ from those reported in psoriasis (37). For example, the association of HLA-C*06, which is consistently associated with psoriasis, is only very weakly associated with PsA. Conversely HLA-B*08, HLA-B*27, HLA-B*38, and HLA-B*39 are the HLA alleles most consistently associated with PsA (38).

HLA-B*27, which is the pre-eminent genetic association with AS, is also consistently associated with PsA but not with psoriasis, thereby indicating different pathogenetic mechanisms in the two conditions despite the obvious disease-in-disease bias for case ascertainment. HLA-B*27 is especially associated with axial skeletal disease and the strength of this association tends to show an inverse relationship with the number of peripheral joints involved (39).

Several other genes involved in the immune response are also associated with PsA. Thus, there are also several other non-HLA genetic associations involving components of the MHC class I antigen processing and presentation pathway: these include *ERAP1* and *ERAP2* (Endoplasmic Reticulum Amino-Peptidase-1 and -2). Others are involved in the innate immune response and the initiation of the immune reaction (e.g. *TLR4* - Toll-Like Receptor 4), or the differentiation and function of CD8+ T-cells (e.g. *RUNX3 -* Runt-related transcription factor 3) (31, 40, 41). Many of these genes are also associated with increased susceptibility to AS, highlighting once again the considerable overlap between these two disorders in terms of their genetic susceptibility (42).

The GWAS era has also highlighted several PsA-associated single nucleotide polymorphisms (SNPs) located in *IL-23A*, *IL-23R*, *IL-12B*, *TYK2* (Tyrosine Kinase 2), and *TRAF3IP2* (which is a downstream target of the IL-17 receptor - IL-17R), implying a pivotal role for the IL-23/IL-17 axis in the pathogenesis of PsA disease pathogenesis (see **Table 1**) (5, 43). A crucial role for IL-17 family is undisputed as the levels of IL-17 and IL-17R were found increased in both psoriatic skin and synovial fluid of patients with PsA (44).

**Table 1:** Genetic variants related to the IL-23/IL-17 axis found associated with PsA through GWAS or consistently identified in targeted analysis studies.

Chromosome	Gene	SNP ID	Odds ratio	Function
1p31.3	*IL-23R*	rs11209026 rs12044149	0.6^#^ 1.4^$^	Th-17 signaling
2q32.2	*STAT4*	rs7540214	N/A	Mediating response to IL-12 and Th-17/Th-1 differentiation
5q33.3	*IL12B*	rs2082412 rs6887695 rs4921482 rs3212227 rs918520	1.4 1.3^@^ 1.4^$^ 1.4^@^ 1.5*	Th-17 and Th-1 differentiation
12q13.3	*IL23A*	rs2020584	N/A	Th-17 signaling
17q21.2	*STAT3*	rs744166		Mediating response to IL-12 and Th-17/Th-1 differentiation
5q33.1	*TNIP1*	rs8177833	1.8*	NFkB signaling
6q21	*TRAF3IP2*	rs33980500 rs13190932	1.7* N/A	Th-17 signaling and NFkB signaling
19p13.2	*TYK2*	rs35251378	1.4*	NFkB, Interferon and Th-17 signaling

*Odds Ratio (OR) from Stuart PE et al. Am J Hum Genet. 2015 3; 97(6): 816–836.

^#^OR from Zhu K et al. Inflamm. Res. 2012; 61**, **1149–1154.

^$^OR from Bowes et al. Nat Commun. 2015 5; 6: 6046.

^@^OR from Filer et al. Arthritis Rheum 2008; 58(12):3705-9.

The contribution of the IL-23/IL-17 axis has greatly advanced our understanding of the pathogenesis of PsA. Th-17 cells produce the pro-inflammatory cytokine IL-17, and all the elements of the Th17 pathway, including MMP3 (Matrix Metalloproteinase 3), CCL1 and CCL20 (Chemokine Ligand 1 and 20) and IL6. The majority of these pro-inflammatory cytokines are upregulated in the blood, synovium, and skin of PsA patients (45, 46). A recent study has identified CXCR6 as a marker for IL-17+CD8+, specialized cells found in the synovial fluid of PsA patients. The presence of CXCR6+IL-17+CD8+ cells in PsA synovium may explain their contribution to the inflammatory environment (47).

IL-23 promotes the survival and expansion of Th-17 cells through its receptor IL23R and the related downstream signaling pathway, which is crucial to Th-17-mediated diseases like PsA. In 2009, a study conducted by Gladman group showed a protective effect for the *rs11209026* SNP in *IL-23R* (encoding the loss-of-function 381Gln allele in the cytoplasmic tail of IL23R) in a Canadian PsA cohort (48). This SNP is also associated with disease severity, while another variant, *rs12044149*, showed an independent peak of association with PsA, after conditioning for the top SNP associated with psoriasis overall (*rs9988642*) (36).

Th17-mediate inflammatory response also involves the signaling adaptor *TRAF3IP2 (*TRAF 3-interacting protein 2), which is downstream of IL-17R. The PsA-associated *TRAF3IP2* variant *rs33980500* alters the binding of the TNF receptor-associated factor 6 (*TRAF6*), which modulates immunoregulatory signals (49, 50).

IL-23 and IL-17 also have major effects on the activation of osteoclasts, which are the main responsible for bone erosion and where the cytokine RANKL (receptor activator of nuclear factor kappa-β ligand) is a critical factor in the promotion of osteoclasts differentiation. RANKL is also expressed by Th-17 lymphocytes and synovial fibroblasts. The dogma that Th17 cells were the primary responsible for bone resorption, was recently challenged in animal studies where cell specific deletion of RANKL indicated that RANKL expression was limited to synovial fibroblasts and B cells (51).

In PsA, bone deposition is crucial as bone resorption. This process arises from the aberrant proliferation, differentiation, and activity of osteoblasts, and several signaling pathways are involved, such as the bone morphogenetic protein (BMP) and the WNT signaling pathway, such as DKK1, and Sclerostin which are relevant for both PsA and AS (52).

The abnormal proliferation of keratinocytes promoting epidermal hyperplasia (53), also emphasizes the predominant functional role of the IL23/IL-17 axis in PsA pathogenesis and in PsA inflammatory cascade.

Lastly, Al Mossawi and colleagues recently demonstrated a marked increase of specific subsets of CD4+ and CD8 + T-cells producing GM-CSF in the blood and synovium of PsA patients. They also demonstrated an increased number of double-positive IL17/GM-CSF for CD4+, CD8+, γδ, and NK (natural killer) cells. The *CSF2* locus encoding GM-CSF, is also strongly associated genetically with PsA. Overall these results suggest a functional link between GM-CSF and the IL-17/IL-23 axis, opening important potential avenues for the treatment of PsA, including those targeting GM-CSF directly (54).

## Cellular Mechanisms in PsA: The Multifaceted Role of IL-17 and IL-23

The contribution of the IL-23/IL-17 axis to the development of PsA will be discussed in this section, highlighting Th-17 biology and the production of a pro-inflammatory *milieu* in the skin and in the synovium, and how this leads to the activation of osteoclasts, which are responsible for bone degradation, and of keratinocytes and neutrophils, which are implicated in the epidermal hyperplasia.

### Cell Activation and Innate Immunity

Inflammation is one of the hallmarks of PsA and activation of the innate immune response is one of the physiological triggers leading to the inflammatory cascade. The transcription factor NF-κB has a major role in this cascade, as it promotes the transcription of several proinflammatory cytokine target genes. Downstream signaling, such as IFNs (Interferons) and TNFα strongly contribute to the immune response in PsA (55).

Several genes involved in these NF-κB pathways show genome-wide significant association with PsA, including *REL* (a subunit of NF-κB), *NOS2*, and *TNFAIP3* (31, 56). The IL-23/IL-17 axis might also influence the activation of the NF-kB pathway in other ways; these include the adaptor protein Act1, which when is phosphorylated binds to TRAF6 to activate the NF-κB activator protein 1 (AP-1), or the CCAAT-enhancer- binding protein (C/EBP) cascade (57).

Interferon signaling also plays a key functional role in the pathogenesis of PsA (58). It is therefore of interest that several interferon-related genes, including *TYK2*, *IFNL1R*, and *PTPN22*, exhibit genome-wide significant association with PsA (59). *TYK2*, for instance, encodes a tyrosine kinase that is involved in initiating IFNα signaling and, as mentioned above, it is also an activator of IL-23R.

TNFα clearly plays a major role in the inflammatory process of PsA as shown by the presence of raised levels of this cytokine at the sites of inflammation and the dramatic responses to treatment with anti-TNF biologics (60). It has a major role in innate immunity, inducing the production of inflammatory chemokines leading to the accumulation of activated T-cells, neutrophils, and monocytes. It is of some interest that significant association for psoriasis and PsA has been found for the SNPs *rs80267959*, *rs1800629*, and *rs361525* at the *TNFA* locus (61).

### Cell Activation and Adaptive Immunity

The pathogenesis of PsA involves components of both the innate and adaptive immune system. Many different cell types are involved in these pathophysiological processes, including T-cells, neutrophils, keratinocytes, and synoviocytes (62).

The master cytokine in the pathogenesis of PsA is IL23. IL-23 is a heterodimer composed of two subunits p19 and p40 subunits, which bind to IL23R. While p19 is unique to IL-23, the p40 subunit is shared with IL-12 (63). The binding of IL-23 to IL23R leads to the recruitment of JAK2 and TYK2 kinases, which are able to activate IL23R (and its cognate IL12RB1), phosphorylate STAT3 (Signal Transducer and Activator of Transcription 3) and induce RORyT (RAR-related orphan receptor gamma) to promote the differentiation, survival, and expansion of Th-17 cells. IL-23 is essential not only in inducing the Th-17 phenotype but also in defining the level of pathogenicity (64).

Th-17 cells are among the possible drivers of the pathogenesis of PsA and the effector molecules they release are able to trigger different target cells such as osteoclasts, macrophages, and synovial fibroblasts. IL-17 is the major cytokine produced by Th-17 cells, gd T cells and other various immune cells. IL-17 A is a homodimer disulfide-linked glycoprotein and it is the most widely studied member of the IL-17 cytokines family, which includes also IL-17B, IL-17C, IL-17D, IL-17E, and IL-17F (65). IL-17A and IL-17 F share 55% of homology and they can form heterodimers, which bind the receptor IL-17R. IL-17R is a dimeric complex consisting of two subunits, IL-17RA and IL-17RC subunits (66). The differential binding affinity of IL-17 for IL-17RA and IL-17RC is still not well define, especially under inflammatory conditions (67).

Th-17 cells differentiate from naïve T-cells depending on a combination of different cytokines along three distinct pathways (68): (i) IL-6 and Transforming Growth Factor-β (TGFβ) (in addition IL-1β and TNFα); (ii) IL-21 and TGFβ; (iii) IL-1β, IL-6, and IL-23 (69). Th-17 cells are characterized by the expression of the transcription factor RORyT with a specific gene signature which in addition to *IL17* also includes *IL6*, *TNF*, *CSF2*, *CCL20*, and *IL23R* (69).

T-cell subsets triple-positive for IL-17, GM-CSF, TNF, or IFN-γ were found increase in PsA synovial tissue. These enriched polyfunctional cells were also found associated with disease activity index (70).

Recently, an elegant study by Taams’ group demonstrated that IL-17+CD8+T cells may contribute to inflammation and disease persistence in PsA. These cells were found increased in patients’ synovium and have a Th-17 resembling transcriptomic profile characterized by high expression of CXCR6 (47).

JAK and STAT are the key signaling pathways transducing the cytokine signals in Th-17 cells (63). Following the inflammatory cascade mediated by IL-1β and IL-23, Th-17 cells release specific pro-inflammatory cytokines such as IL-17, GM-CSF, and IL-22. The function of Th-17 cells is tightly dependent on the balance of the effector molecules they produced, such as IL-23 and TGF-β, and on those cytokines promoting cell differentiation and maintenance (IL-23).

The effects of IL-23 on bone are conflicting. IL-23, in a Th-17 independent way, up-regulates the expression of the nuclear factor kappa-β ligand RANKL-receptor RANK in osteoclast precursors (from monocyte lineage cells), favoring osteoclast differentiation and proliferation (64, 71). IL-23 also induces the production of GM-CSF, which is an inhibitor of the differentiation of osteoclasts, thus limiting bone resorption (72). RANKL expression is also found in synovial fibroblasts where its deletion plays a key role in bone erosion (51).

Neoangiogenesis also appears to have an important part in the pathogenesis of PsA; new blood vessels are prominent in the synovial histology of the condition. It appears that the IL-17/IL-23 axis might play a role in the angiogenic process. As we have described, IL-17 produced by Th-17 cells up-regulates proinflammatory cytokines and prompts the recruitment of neutrophils and macrophages and endothelial cell migration. Further, keratinocytes, stimulated by IL-17 ligands, start to differentiate and proliferate aberrantly, producing proinflammatory adenosine monophosphate (AMP), chemokines, and angiogenic factors such as vascular endothelial growth factor (VEGF) (73).

IL-23 can also promote epidermal hyperplasia activating the proliferation of keratinocytes (increasing the expression of keratin 16) and by acting synergistically with IL-17 promotes the recruitment of neutrophils and the infiltration of IL-22 and IL-17 producing-cells into the lesioned skin (74). The response of keratinocytes and endothelial cells among others to IL-17 and IL-22 stimulation leads to the upregulation of chemokines such as CXCL1 and CCL20, pro-inflammatory cytokines, and anti‐microbial peptides, such as LL‐37 and β‐defensins. IL-17 and IL-22 both promote keratinocyte proliferation and the recruitment of macrophages and neutrophils; they also decrease the expression of adhesion molecules (i.e. selectins and integrins) thus favoring the disruption of the skin barrier. IL-17 can also stimulate the expression of endothelial markers such as P- and E-selectins and adhesion molecules, including ICAM-1 (Intracellular Adhesion Molecule 1) and VCAM-1 (Vascular Cellular Adhesion Molecule 1), which enhances the mobilization of neutrophils (75).

Lastly, the expression of IL-17 and IL-23 is increased in the synovium (76, 77). Gene expression analysis of PsA synovium reveals a gene signature closer to PsA skin than to rheumatoid synovium (78). The recruitment of pathogenic IL-23/IL-17- producing CD4+ T-cells has been demonstrated to be higher in the joints, while the IL-17/IL-22 producing CD4+ T-cells are strongly detected in the skin and in the circulation (79, 80).

## Therapeutic Approaches in PsA

Since the development of biologic therapies, the ultimate target for the treatment of any patient with psoriasis and/or PsA in modern times is complete remission (81). Initially these targeting TNFα were used with great effect but much effort has also been made to develop biological drugs targeting the IL-23/IL-17 axis. This axis, as specified previously explained, offers several plausible drug targets, such as the p40 subunit of the IL-23/IL-12 receptor, the p19 subunit of IL-23R, IL-17A and its specific receptor IL-17R (82).

In the treatment of PsA non-steroidal anti-inflammatory drugs (NSAID) and synthetic disease modifying antirheumatic drugs [sDMARDs, such as methotrexate (MTX), leflunomide, and sulfasalazine] remain the first-line therapies but biological molecules (bDMARDs) and targeted synthetic DMARDs (tsDMARDs) are used if therapy with NSAID and DMARDs fails to control the disease adequately.

Biologics (such as etanercept, infliximab, adalimumab, golimumab, certolizumab, ustekinumab, and secukinumab) or synthetic drugs (apremilast, tofacitinib and ixekizumab) are given in specific circumstances (62). The different drugs used in PsA (and in other conditions) with their mechanism of action are shown in **Table 2**.

**Table 2:** Overview of the current treatments in PsA and related mechanism of action.

Category	Molecule	Mechanism of action	Approval and use	ACR20 in PsA (%)
sDMARDs	Methotrexate	Anti-metabolic	RA, Ps, and PsA	32–40
	Leflunomide	Inhibitor of pyrimidine synthesis	RA, Ps, and PsA	34
bDMARDs	Etanercept	TNFα blocker	AS, RA, and Ps	60–65
	Infliximab	TNFα blocker	AS, RA, Ps, and PsA	65
	Adalimumab	TNFα blocker	Ps and PsA	58
	Golimumab	TNFα blocker	PsA, AS, and RA	76
	Certolizumab	TNFα blocker	RA and Ps	52-63
	Brodalumab	IL-17RA inhibitor	Ps and PsA	39
	Ustekinumab	IL-12/IL-23 inhibitor	Ps and PsA	42–50
	Secukinumab	IL-17A inhibitor	AS, Ps, and PsA	54
	Guselkumab	IL-23 inhibitor	Ps, PsA	58
	Ixekizumab	IL-17A inhibitor	Ps, and PsA	60
tsDMARDs	Apremilast	PDE4 inhibitor	Ps and PsA	31
	Tofacitinib	JAK-1/3 inhibitor	PsA	50–53

sDMARDS, synthethic disease modifying anti-rheumatic drug; bDMARDs, biologic disease modifying anti-rheumatic drug; tsDMARDS, targeted synthetic disease modifying drug; ACR20, American College of Rheumatology 20 response; Ps, psoriasis; IL, interleukin; PDE4, phosphodiesterase type 4; TNFα, tumor necrosis factor α (15, 62, 82, 83).

A combination of two or more of these drugs is often administered in complex immunological diseases like PsA, and often results in increased efficacy compared with monotherapy (83).

In recent few years, a number of guidelines have been developed for the clinical assessment and management of PsA; these include recommendations from GRAPPA, EULAR (European League Against Rheumatology) and ACR/NPF (American College of Rheumatology/National Psoriasis Foundation) (84, 85). For instance, EULAR recommends NSAID and local glucocorticoid injections, especially for enthesitis, in the early phase of the disease. If adverse prognostic factors are present or treatment fails, the administration of sDMARDs, such as MTX (or alternatively leflunomide or sulfasalazine) is recommended (86). If these fail to control the disease adequately or are poorly tolerated the administration of TNF inhibitors should be considered either in combination with DMARDs or not. Biologics should also be used in those with prominent axial disease or severe enthesitis. The continued use of TNF inhibitors should be evaluated carefully according to the patient’s response and a switch to alternative biologics may be considered either where there is no initial benefit (primary failure) or where the response is lost after a period of time (secondary failure) perhaps due to the host generation of antibodies to the biologic (81).

### Targeting the IL-17/IL-23 Pathway

**Figure 1:**
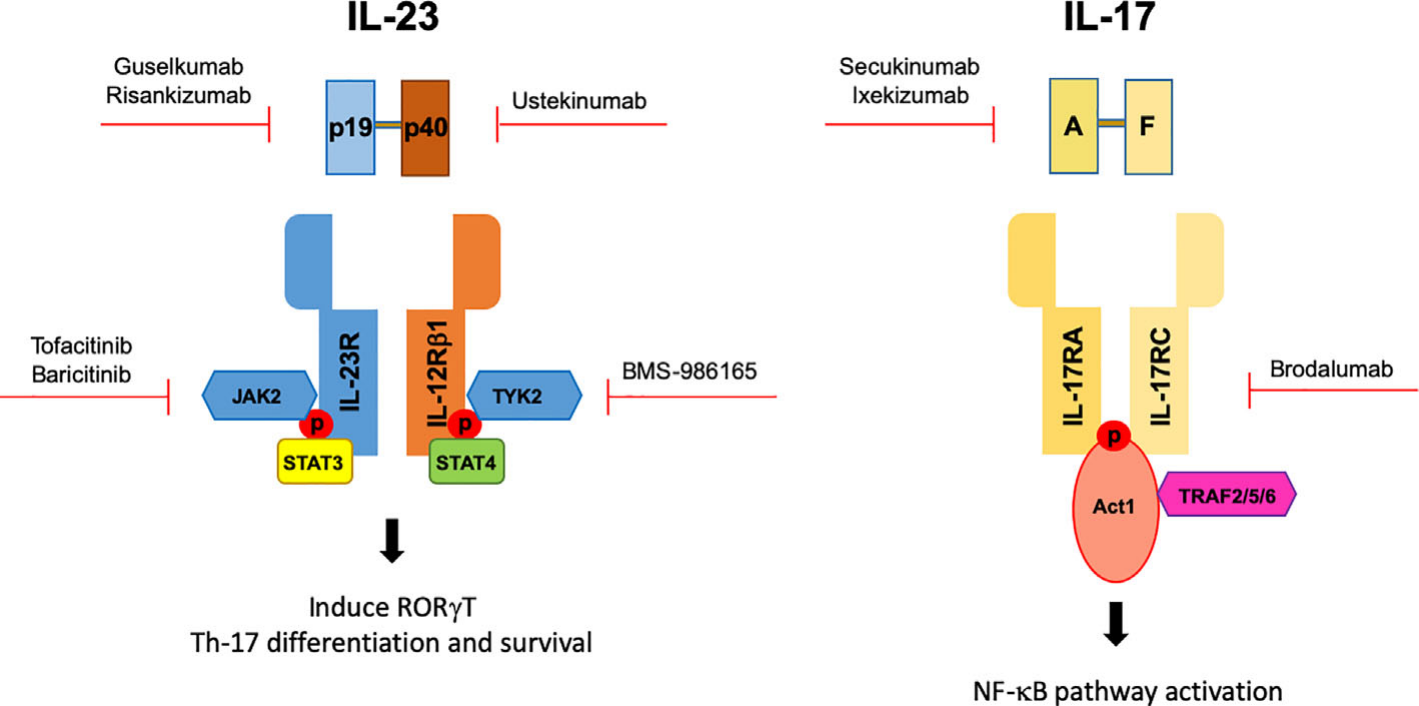
Genetics studies allowed the identification of the IL-23/IL-17 axis having a crucial functional role in PsA pathogenesis. Genetics lead to the development of biological drugs targeting the IL-23/IL-17 axis in PsA. Red arrow indicates the target of different biologics.

The IL-17/IL-23 pathway and Th-17 cells have become a favorite target in PsA in recent years. For this purpose, several biological drugs have been developed (**Figure 1**). Ustekinumab, which is a monoclonal antibody targeting p40 subunit of IL-12/IL-23 has been used for skin and nail psoriasis but also for peripheral PsA in those patients who not respond not to DMARDs (87).

Two large clinical trials, PHOENIX 1 and 2, assessed the efficacy of ustekinumab in psoriasis patients (88) while PSUMMIT-1 and -2 examined its efficacy and safety in PsA (89). *Post-hoc* analyses confirmed the efficacy of ustekinumab not only on skin but also in improving PsA rheumatological manifestations and radiographic progression (90). The efficacy of ustekinumab in axial involvement for PsA or in axial SpA is believed to be marginal and the development programs have been thus discontinued. However, the final word on the efficacy of IL23 blockers in SpA remains uncertain as recent data on guselkumab showed efficacy on patient reported outcomes for PsA axial manifestations (91) and a recent phase IV study on ustekinumab reported that this drug is frequently used in patients with axial PsA (92). Inhibition of IL-17A has been achieved using secukinumab, a human monoclonal antibody targeting IL-17A, with efficacy in both PsA and ankylosing spondylitis (93, 94). In the treatment of PsA secukinumab is effective for dactylitis, enthesitis, skin and nail lesions, but its effects on joint disease is rather less, as shown in FUTURE 2 and 3 trials (95).

Targeting either IL-17 or its receptor in PsA patients include besides secukinumab, also ixekizumab and brodalumab. The efficacy of ixekizumab (also targeting IL-17A) was demonstrated in reducing active disease and radiologic progression in joints, as well as fulfilling the PsA criteria of skin response, as demonstrated in the head-to-head SPIRIT study (96). Furthermore, brodalumab, which is a human monoclonal antibody human anti-IL17RA, a pan inhibitor of IL-17A, IL-17F, and IL-25 is currently used in the treatment of psoriasis where shows a complete clearance of moderate-to-severe psoriasis (97). Brodalumab efficacy and safety was also assessed in PsA patients (98).

DISCOVER-1 and -2, a double-blind, randomized, placebo-controlled phase 3 trials proved the efficacy of Guselkumab a human monoclonal antibody specifically binding the p19 subunit of IL-23. The study has shown a substantial improvement in biological naïve patients with active disease, in particular in decreasing IL-17A, IL-17F, and CRP serum levels by week 16 achieving Psoriasis Score and Severity Index, PASI75 (99, 100).

Overall, following the blockade of the IL-23/IL-17 axis, clinical trials for Ps and PsA showed a good amelioration of the skin lesions while the joint response was much lower. A larger percentage of patients achieved the PASI75, PASI90 and PASI100 compared to proportion of patients fulfilling the ACR20, ACR50, or ACR70 criteria of response (101).

## Discussion and Concluding Remarks: How Genetics Might Be Crucial In Identifying Credible Therapeutic Targets

PsA is a complex polygenic disease with a genetic contribution that overlaps with other related conditions such as psoriasis, AS and IBD. Genetic variants associated with a specific disorder have the power to highlight genes or pathways that may contain credible targets for drug therapy. This is well exemplified for the IL-17/IL-23 axis and Th-17 cells with the development of biological drugs blocking IL-17 (i.e Secukinumab in AS), or IL-23 (i.e. Ustekinumab in psoriasis/PsA). Unfortunately, the process is challenging (102).

The first crucial point following a GWAS is to accurately assign associated SNPs to the genes they regulate in order to define credible pathways. For this purpose, several experimental functional assays have been developed. Functional disease-associated SNPs may affect the binding of transcription factors or the enrichment of regulatory markers: this is currently evaluated with *in vitro* Electrophoretic Mobility Shift Assays and *ex vivo* with Chromatin ImmunoPrecipitation). SNPs may have an effect on gene expression (evaluated with expression quantitative trait loci, eQTL studies) or on chromosome looping (assessed *via* chromosome conformation assays). Genome editing techniques are performed to define the consequences of harboring a risk variant on cellular function.

Second, these experiments must be performed considering cell-specificity (i.e. specific tissue or cell type) and condition-specificity (i.e. different stimulatory conditions) to provide significant insights into the pathogenesis of a specific disease and develop targeted therapies.

This approach will increase our understanding in defining credible pathways, specific genes, and cell populations for targeted therapy. The better molecular stratification we can achieve (for instance, patients with enrichment of associated SNPs in the IL‐23/IL‐17 pathway may respond more effectively to secukinumab or ustekinumab), the better the design of personalized therapeutic strategy will be. The final goal will be an advanced use of SNPs as pharmacogenetic markers, in order to define credible pathways to target and predict response to biological therapy (i.e. HLA-C*06 as a pharmacogenetic marker in response to Ustekinumab) (103).

## Author Contributions

MV, VH, BW, and CS conceived the manuscript. MV, VH, CD, MC, BW, and CS drafted the manuscript, and all the authors revised the final version prior to submission. All authors contributed to the article and approved the submitted version.

## Funding

CS is funded by Ministero degli Esteri (Italia), grant ITALY–CHINA - SCIENCE AND TECHNOLOGY COOPERATION: PGR05455. MV is funded by Versus Arthritis#21428, CD is funded by Versus Arthritis#22198.

## Conflict of Interest

The authors declare that the research was conducted in the absence of any commercial or financial relationships that could be construed as a potential conflict of interest.
